# Serosurvey for *Brucella* spp. and *Coxiella burnetii* in animals on Caribbean islands

**DOI:** 10.1002/vms3.214

**Published:** 2019-11-14

**Authors:** Jason W. Johnson, Helene Lucas, Sharon King, Tyler Caron, Chengming Wang, Patrick J. Kelly

**Affiliations:** ^1^ College of Veterinary Medicine Lincoln Memorial University Harrogate TN USA; ^2^ Department of Clinical Studies Ross University School of Veterinary Medicine St Kitts West Indies; ^3^ Department of Pathobiology College of Veterinary Medicine Auburn University Auburn AL USA

**Keywords:** *Brucella*, Caribbean, *Coxiella*, serosurvey

## Abstract

To determine the prevalence of antibodies to *Brucella melitensis, Brucella abortus* and *Coxiella burnetii* in animals on Caribbean islands we obtained sera from convenience samples of cattle (C), sheep (S), goats (G) and cats (F) from Dominica (C, S, G), Grenada (C, S, G), Montserrat (C, S, G), Puerto Rico (C), Nevis (C, S, G), St Kitts (C, S, G, F) and St Lucia (C, G). The sera were tested for antibodies against the *Brucella* spp. using commercial ELISA kits. Some sera were also tested at 1/80 for antibodies to *C. burnetii* using an indirect fluorescent antibody test. Positive sera were also tested at 1/640. None of 599 cattle, 462 sheep or 434 goats were positive in the *Brucella* ELISAs. None of 230 cattle had antibodies against *C.* *burnetii*, but one of 299 sheep was positive at 1/80 (Dominica – 1/54, 2%, 95% CI (0%–5.6%)), as were two of 314 goats, at 1/80 (Grenada – 1/53, 2%, 95% CI (0%–7.5%)) and 1/640 (St Kitts − 1/18, 5.6%, 95% CI (0%–16.7%)), and one of 34 cats, at 1/80 (St Kitts − 1/34; 3%, 95% CI (0%–8.8%)). Our data suggests that there is a very low prevalence or absence of *B. melitensis* and *B. abortus* on Caribbean islands. *Coxiella burnetii*, however, is present but it appears to be present on only some islands and then only at low levels. Overall, there appears to be a low threat to human and animal health from these organisms in the Caribbean.

## Graphical abstract

A serosurvey of animals from Caribbean islands showed little evidence of exposure to *Coxiella burnetii* and *Brucella* spp., the agents of Q fever and brucellosis in animals and people, respectively. The findings indicate that there appears to be a low threat to human and animal health from these organisms in the region.

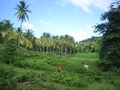

## 1 INTRODUCTION

*Brucella melitensis*, *Brucella abortus* and *Coxiella burnetii* are important zoonotic agents that occur almost worldwide (Babudieri, 1959; Corbel, 1997). The *Brucella* spp. are Gram‐negative facultative intracellular bacteria that pose serious health threats to animals and people and are classified by the World Health Organization as a class B2 agent that is reportable in most countries. In domestic animals, they can cause abortions, delayed pregnancy and decreased milk production, which result in significant economic losses (Seleem, Boyle, & Sriranganathan, 2010). In people, they generally cause nonspecific signs such as fever, fatigue, anorexia, sweating and headache, but sometimes there are serious sequelae including encephalitis, endocarditis and abortion (Godfroid et al., 2005; Seleem et al., 2010). While many first world countries have instituted control and eradication programs for *Brucella* spp., infections are still common in developing countries in Africa, Asia, the Middle East, Mediterranean Basin and Central and South America (Seleem et al., 2010). Current literature places the Caribbean in the high‐risk category, however, this categorization may be unfounded considering the lack of research (Godfroid et al., 2005). There is limited data on *Brucella* spp. in livestock in the Caribbean with Puerto Rico, Barbados, Saint Kitts and Nevis reportedly free of the organisms (Corbel, 1997; Givens, 2006; Stone et al., 2012). Infections have, however, been reported with *B.* *abortus* in Cuba, the Dominican Republic, Haiti and Trinidad (Corbel, 1997; Fosgate, Adesiyun, Hird, Hietala, & Ryan, 2002; Peraza, Valdes, & Fonseca, 1998). While serological evidence of *B. melitensis* in sheep and goats on Saint Croix was presented in 1993 (Ahl, Bartlett, & Frerichs, 1993) the organism was subsequently reported to be eradicated (Givens, 2006). In a recent report, sheep and goats from Cariacou (2.1%; 13/362) and Grenada (4.5%; 10/221) (Stone et al., 2012) and cattle (6%; 9/150) from Grenada (Chikweto et al., 2013) were positive for antibodies to *Brucella* spp. A brucellosis control program in Trinidad led to large scale culling of infected water buffalo in Trinidad between 1999 and 2009 (Fosgate, Diptee, Ramnanan, & Adesiyun, 2011) and a recent survey of milk from 92 dairy cattle farms failed to identify antibodies to *abortus* indicating the organism was unlikely to be circulating in the dairy cattle population (Morris et al., 2018).

*Coxiella burnetii* is also a Gram‐negative bacterium and can infect a wide variety of ticks, rodents, birds, wild and domestic mammals, and people (Kelly, 2005). Domestic ruminants are the major reservoirs for *C. burnetii* infections in people (Maurin & Raoult, 1999). Although cattle, sheep and goats may remain chronically infected for weeks to years, the majority of infections are subclinical. They can, however, shed large numbers of organisms in the amniotic fluid, placenta (>10^9^/g), foetal membranes, faeces and urine and contact with infected domestic livestock is a major risk factor for humans developing Q fever (El‐Mahallawy et al., 2016; Thomas et al., 1995). Cats have also been implicated in a number of outbreaks of human Q fever and they have been shown to be important reservoirs of infection for people in some areas (Marie, Durant, Williams, Mintz, & Wang, 1988).

Infections with *C. burnetii* occur principally by inhalation of infected aerosols of periparturient fluids with only a single organism being sufficient to cause infection (Maurin & Raoult, 1999). The organism is extremely resistant to desiccation, low or high pH, disinfectants and ultraviolet radiation and may remain infective in aerosols for up to 2 weeks and in the soil for as long as 5 months (Scott & Williams, 1990). In people, acute infections are heavily underdiagnosed as they are most often asymptomatic; they can also resemble a mild flu‐like illness with fever, headache and myalgia that resolves spontaneously in a week (Maurin & Raoult, 1999). In some cases disease is more severe and there may be atypical pneumonia (Marrie et al., 1981). In patients who do not eliminate *C. burnetii*, chronic infections may occur and present with signs including endocarditis, chronic hepatitis and osteomyelitis which are difficult to treat and have a poor prognosis (Maurin & Raoult, 1999).

Although the Caribbean Islands have strong agricultural bases and populations of domestic animals, there is little data on Q fever in the region. A serosurvey has shown Q fever was not prevalent in people in Jamaica (Grant, 1961) and a study on dogs belonging to the French military failed to detect reactive antibodies in sera from seven animals from Martinique (Boni, Davoust, Tissot‐Dupont, & Raoult, 1998). A study in Trinidad showed pigs were infected (11%) but not sheep and goats (Adesiyun & Cazabon, 1996). In Grenada and Carriacou, all 362 sheep and 220 goats studied were negative by ELISA (Stone et al., 2012).

To provide more information on the status of *Brucella* spp. and *C. burnetii* infections in the Caribbean we carried out serosurveys on convenience samples of animals from seven Caribbean islands. Our results are described in this report.

## 2 MATERIALS AND METHODS

Approval for this study was obtained from the IACUC of Ross University School of Veterinary Medicine, St. Kitts. Convenience samples of sera from apparently healthy adult cattle, sheep, goats and cats were obtained as described previously (Kelly et al., 2010; Moura, Kelly, Krecek, & Dubey, 2007) and stored at −20°C until use. Antibodies to *B. abortus* in the cattle sera/plasma were detected with the Porquier ELISA Brucellosis Individual and Pool Serum Screening Kit according to the manufacturer's instructions (Institut Pourquier). Two sets of positive and negative controls were supplied in the kit by the manufacturer. In addition, we used six positive control sera from naturally infected bubaline species in Trinidad (kindly supplied by A.A. Adesiyun). Antibodies to *B. melitensis* in serum and plasma from sheep and goats were detected using the Pourquier ELISA Sheep and Goat Brucellosis Screening Kit according to the manufacturer's instructions (Institut Pourquier). Two sets of positive and negative controls were provided by the manufacturer.

Some of the sera were also tested for IgG antibodies to phase II *C. burnetii* using an indirect fluorescent antibody test as described previously (Kelly, Matthewman, Mason, & Raoult, 1993). Plasma were screened initially at a titre of 1/80 in PBS using appropriate fluorescein labelled antisera (H + L) and positive samples were screened again at 1/640. Positive control sera and the antigen were supplied by the Centers for Disease Control and Prevention (Atlanta).

Using bootstrapping, the 95% confidence intervals were calculated with the Statistics Base option in IBM SPSS Statistics for Windows, version 24.0 (IBM Corp.).

## 3 RESULTS

Test sera from 1,495 animals, comprising 599 cattle, 462 sheep and 434 goats, were obtained from seven Caribbean islands and tested in the *Brucella* ELISAs (Table 1). While positive control sera gave significant ELISA results, all test sera were clearly negative for reactive antibodies. In the *C. burnetii* IFA tests which were conducted on some of the sera, none of the cattle we studied had reactive antibodies (Dominica – 0/83; Grenada – 0/4; Montserrat – 0/14; Nevis – 0/38; St Kitts ‐ 0/55, Puerto Rico 0/26 and St Lucia 0/72) while only one sheep was positive at 1/80 (Dominica – 1/54 (2%, 95% CI (0%–5.6%)); Grenada – 0/86; Montserrat – 0/53; Nevis – 0/101; St Kitts – 0/5). Two goats were seropositive, one from Grenada at 1/80 and 1 from St Kitts at 1/640 (Dominica – 0/65; Grenada – 1/53 (2%, 95% CI (0%–7.5%)); Montserrat – 0/27; Nevis – 0/126; St Kitts ‐ 1/18 (5.6%, 95% CI (0%–16.7%)) and St Lucia 0/18).

**Table 1:** Sources of the sera used in the study

Island	Sites	Cattle	Sheep	Goats	Cats
Dominica	7	95	57	137	0
St Kitts	2	86	47	25	34
Nevis	12	45	132	146	0
Montserrat	5	12	139	29	0
Grenada	5	4	87	97	0
St Lucia	ND	173	0	0	0
Puerto Rico	4	184	0	0	0
Total	35	599	462	434	34

One of the 34 (3%, 95% CI (0%–8.8%)) feral cat sera from St Kitts had antibodies to C. burnetii at 1/80.

## 4 DISCUSSION

In our experiments, we failed to find evidence of infections with *B. abortus* or *B. melitensis* in domestic ruminants from seven Caribbean islands, mainly Dominica, Montserrat, Nevis, Puerto Rico, Grenada, St. Kitts and St. Lucia. Our results provide some firm data in support of earlier anecdotal reports that Puerto Rico, St Kitts and Nevis are free of infections (Corbel, 1997; Givens, 2006). They are also the first to provide data on *Brucella* spp. on three other islands, mainly Dominica, St Lucia, and Montserrat, where we also failed to find any evidence of infections. However, although we did not detect positive cattle, goats or sheep on Grenada, 4.5% of the sheep and 6% of cattle examined in recent studies (Chikweto et al., 2013; Stone et al., 2012) were found positive using a similar commercial ELISA test (SAVNOVIR® Brucella‐Ab‐IELISA, Svanova biotech Ab). The positive sheep they found on Grenada (7/163) came from three parishes where we also sampled sheep (65) which we found were all negative. While bloods were collected at different times in the studies (March 2008 vs. December 2009–2010) and infections might have been introduced in the intervening period, it is also possible that infections on Grenada are focally distributed and our sample size was inadequate for their detection. The positive cattle in Grenada (9/150) came from three of the six parishes surveyed (Chikweto et al., 2013). Although we only surveyed four cattle from Grenada they were from one of the negative parishes in the above study. Additional studies are indicated on the other islands we studied to determine if there is a low prevalence of infection or *Brucella* spp. are indeed absent.

Overall, our results and the data available indicate infections with *Brucella* spp. are probably at a low level or absent on many islands in the Caribbean and, if brucellosis occurs, it may still be localized to only certain areas. Studies on larger populations are needed to clarify the situation. As there are no wildlife reservoirs on the islands, apart from Trinidad where water buffalo may still be infected (Fosgate et al., 2011), it seems maintaining or gaining a disease free status and the associated health and economic benefits will depend on identifying and eradicating foci of infection and preventing the disease being imported. The latter is important as brucellosis does occur in the Caribbean, albeit at low levels in at least Grenada and Cariacou (Stone et al., 2012), and is endemic in many surrounding countries in central and South America (Corbel, 1997). Strict import regulations should be in place on islands where there is no evidence of brucellosis to prevent the introduction of infected animals or their products.

Although our sample size was relatively small, we found evidence of *C. burnetii* infections being relatively widespread, occurring on at least three of the seven Caribbean islands studied. It is noteworthy that, although *C. burnetii* occurs worldwide, it is absent in New Zealand (Hilbink, Penrose, Kovacova, & Kazar, 1993) and might then also not be present on some of the Caribbean islands.

While the organism might be relatively widespread in the Caribbean, our results indicate the seroprevalences in domestic ruminants and cats, the major sources of infection for people, are low. Studies in other countries where people also live in close association with livestock have shown far higher prevalences of antibodies to *C. burnetii* in domestic ruminants. For example, in Africa reported seroprevalences have varied from 2% in Malawi (Staley, Myburgh, & Chaparro, 1989) to 75% in Zimbabwe (Kelly et al., 1993). Similarly, the prevalence we found in cats from St Kitts (3%) was considerably lower than that recently reported for stray cats from Japan (42%) (Komiya et al., 2003) and the United Kingdom (62%) (Meredith, Cleaveland, Denwood, Brown, & Shaw, 2015). Possible reasons for the lower prevalences in the Caribbean islands might relate to the fact that livestock are often left to roam freely in uninhabited areas and thus people are less likely to be exposed to periparturient fluids. Also there is lack of efficient wildlife vectors and species of ticks required to maintain infections (Babudieri, 1959; Kelly, 2005).

The low prevalences we found in our study suggest the threat posed by *C. burnetii* to human and animal health in the Caribbean is also low. This is consistent with a previous report from Jamaica where 3/304 sera collected between 1952 and 1956 were seropositive but at very low and doubtful titres (Grant, 1961). No sera collected in 1960 were positive. The test used in the report was the complement fixation test that is less sensitive than the indirect IFA used in our study (Maurin & Raoult, 1999). More recently, an ELISA study revealed antibodies to *C. burnetii* in 2.3% of 442 pregnant women from 10 Caribbean countries (Wood et al., 2014). Seropositive women were identified on St Kitts (13.6% ± 10.1), Montserrat (6.6% ± 12.7), Antigua–Barbuda (5.3% ± 6.6) and Jamaica (2.1 ± 4.1) but not from Belize, Bermuda, Dominica, Grenada, St Lucia and St Vincent‐Grenadines. Unfortunately, there was no travel history for the women and it is not possible to determine if they were exposed in the Caribbean or elsewhere in the world.

In conclusion, our study has shown infections with *Brucella* spp. in domestic ruminants in the Caribbean are probably uncommon, as are infections of domestic ruminants and cats with *C. burnetii*. The apparent lack of reservoirs of infection would suggest people are also at low risk of infection with these organisms.

## CONFLICT OF INTEREST

The authors declare there have been no conflict of interests.

## ETHICAL STATEMENT

The authors confirm that the ethical policies of the journal, as noted on the journal's author guidelines page, have been adhered to and the appropriate ethical review committee approval has been received. The US National Research Council's guidelines for the Care and Use of Laboratory Animals were followed.
